# Changes in the Perceptions of Oral Symptoms Due to COVID-19 in Korean Adolescents

**DOI:** 10.3390/dj12010010

**Published:** 2023-12-30

**Authors:** Sun-Kyoung Lee, Jeong-Min Seong

**Affiliations:** 1 Department of Dental Technology, Kyungdong University, 815, Munmak-eup, Wonju 26495, Gangwon-do, Republic of Korea; oksk3737@kduniy.ac.kr; 2 Department of Dental Hygiene, Kangwon National University, 346, Dogye-eup, Samcheok-si 25913, Gangwon-do, Republic of Korea

**Keywords:** COVID-19, Republic of Korea, adolescents, oral symptoms, subjective health perception

## Abstract

This study investigated the oral symptoms and subjective health perceptions of Korean adolescents due to COVID-19. Data from the 17th Korea Youth Risk Behavior Survey (KYRBS; 2021) were analyzed, and 54,848 Korean middle and high school students were included in the study. Frequency, cross-tabulation, and logistic regression analyses were performed using IBM SPSS (v. 22.0; IBM Corp., Armonk, NY, USA). Statistical significance was set at *p* < 0.05. The survey results on subjective health perception showed that 64.8% considered themselves “healthy”, 26.1% rated themselves “moderate” in health, and 9.26% rated themselves “unhealthy.” When asked about brushing their teeth after lunch at school, students answered “no” more frequently than “yes”. Participants provided significantly different responses to questions related to receiving “sealant treatment”, “having broken teeth”, “experiencing tooth pain when eating”, “having throbbing tooth pain”, and “having pain and bleeding in gums”. Regarding the relationship between oral symptoms and subjective health perceptions due to COVID-19, students perceived themselves as healthy when they had no throbbing tooth pain, risk of pain, bleeding in the gums, or symptoms of toothache when eating. Results reveal a relationship between oral symptoms and subjective health perceptions due to COVID-19. Thus, appropriate oral health management for adolescents is needed in response to the COVID-related changes.

## 1. Introduction

Adolescence is a transitional period from childhood to adulthood during which children undergo significant physical, emotional, and social changes [1]. Adopting a healthy lifestyle during adolescence is easier and has a long-lasting impact on overall health in adulthood [2]. As health behaviors in adolescents have received more attention, recent studies have focused on factors influencing oral health in adolescents [3]. In the Republic of Korea, the Korean National Health and Nutrition Examination Survey, Korean National Oral Health Status Survey, and Korea Youth Risk Behavior Web-Based Survey (KYRBS) have been conducted to study health behaviors in adolescents and include oral health-related questions, making it possible to determine their oral health status.

The COVID-19 pandemic has significantly changed people’s daily lives worldwide. Governments have implemented social distancing measures and mandated or recommended the use of masks, while schools have conducted online classes. These environmental changes have impacted the oral health of many individuals [4]. Kim [5] reported a higher likelihood of adolescents experiencing gum pain and bleeding since the onset of COVID-19, while Park et al. [4], in a study on college students, noted that teeth clenching incidence increased in its wake. One typical intraoral symptom related to COVID-19 is dysgeusia; however, symptoms vary depending on individual conditions, including the presence of ulcers, erosions, vesicles, pustules, macules, papules, plaques, and erythema [6]. COVID-19 and long COVID can negatively affect systemic and mental health as well as lifestyle factors, including oral hygiene.

Subjective health perception is measured based on how individuals perceive their own health status. Various scales are used to measure the concept of subjective health perception, including indicators related to quality of life, such as health satisfaction, perception of status in functional limitation, and avoidance of interpersonal relationships due to health problems. Subjective scales for oral health include the self-assessment of teeth and gums.

Numerous studies on the relationship between COVID-19 and oral health have been published thus far, including those on the effects of COVID-19 on dental visits in adults [7], oral health behaviors and symptoms in college students [4], and the effects of COVID-19 on dental consultations across all age groups [8]. Despite the significant global disruption caused by COVID-19, there is a paucity of studies on the relationship between oral symptoms and the subjective health perceptions of adolescents in its wake. Therefore, this study aimed to investigate the oral symptoms and subjective health perceptions of adolescents since the onset of COVID-19 and to provide guidance for developing an oral management program for adolescents by efficiently dealing with the changes brought about by the COVID-19 pandemic.

## 2. Materials and Methods

### 2.1. Study Participants

This study used data from the 17th KYRBS (2021) [9]. Data were provided by the Korea Disease Control and Prevention Agency (KDCPA) according to the request procedure for acquiring KYRBS raw data. A total of 54,848 middle and high school students in the Republic of Korea were included as participants. The KYRBS is an anonymous self-report online survey conducted on 7th–12th graders in the Republic of Korea to investigate their health status, including their smoking, drinking, obesity, diet, and physical activity details. It is a government-approved statistical survey conducted annually since 2005. Its purpose is to generate health behavior statistics among the Korean youth, which are then utilized for the planning and evaluation of health policies and health-promoting projects for adolescents. The target population of the 17th KYRBS (2021) was defined as current middle and high school students nationwide as of April 2021.

For the sampling frame, data from Korean middle and high schools as of April 2020 were used. High schools were categorized into general high schools (general high schools, autonomous high schools, science high schools, foreign language high schools, arts high schools, and physical education high schools) and specialized high schools (specialized high schools and meister high schools). The sample selection process can be divided into population stratification, sample allocation, and sample extraction stages. In the population stratification stage, the population was divided into 117 strata using 39 regions and school levels (middle school, general high school, and specialized high school) as stratification variables. Regarding the regions, si, gun, and gu within 17 cities and provinces (including Sejong City since 2015) were classified into large cities, small and medium-sized cities, and gun, which were then subsequently classified into 39 regions in consideration of geographical accessibility, number of schools and population, living environment, smoking rate, and drinking rate. In the sample allocation stage, the sample size was allocated to 400 middle schools and 400 high schools, and five middle and high schools each were initially allocated to each of the 17 regions. Proportional allocation was employed to match the population composition ratio and the sample composition ratio by stratification variable, and the sample schools were allocated based on the city/province, city size (large city, small and medium-sized city, and gun), region, middle school (male, female, and co-ed), high school (male, female, and co-ed), and general high school/specialized high school. For sample extraction, stratified cluster sampling was used, with schools as the first-stage sampling unit and classes as the second-stage sampling unit. In the first-stage sampling, sample schools were selected using permanent random sampling within each stratum. In the second-stage extraction, one class per grade was randomly selected from the selected sample schools. All students in the selected sample classes were surveyed, and long-term absentees as well as special-needs children and students with decoding difficulty who could not participate in the survey independently were excluded from the sample student group.

The following considerations were made for the 2021 sampling. First, a sample school with fewer than 50 students, scheduled for closure, or in an extended period of closure was replaced with a school on the same stratum that was geographically nearby and had similar characteristics. Second, there was a risk of under-sampling of specialized high schools due to the small number of such schools, but weighting was applied during population estimation. Third, when selecting sample classes, one class was chosen while considering different types of classes, such as liberal arts, natural sciences, college preparatory, and vocational classes, for high schools.

### 2.2. Study Tools

From 2018, a rotational survey system has been implemented to reflect diverse needs for the indices without marked changes in the total number of items. The items are categorized as essential (surveyed every year) and rotational (surveyed every three years) based on importance, and an in-depth survey is conducted including rotational items for each domain. In 2021, smoking, drinking, injury, safety awareness, and sex behavior domains were surveyed. Rotational items included place of smoking, method of cigarette purchase, awareness of smoking cessation advertisements, exposure to cigarette advertisements, and smoking prevention and smoking cessation education experiences for the smoking domain. For the drinking domain, exposure to alcoholic beverage advertisements (to investigate drinking-promoting environments) and low-alcohol carbonated drink intake were newly added, and school drinking prevention education, place of drinking, indirect harms from drinking, and permission to drink at home were surveyed. For the injury and safety awareness domain, items about use of a helmet for motorcycle/bicycle, history of injury from earphones or cellphones, and history of injury at school were added. For the sex behavior domain, time of sex initiation, sex after drinking, and pregnancy were used as rotational items.

In 2021, items about mental health (loneliness, seven-item Generalized Anxiety Disorder scale [GAD-7]), personal hygiene (washing hands with soap and water after returning home), and health equity (changes in household finances due to COVID-19) were surveyed in consideration of the COVID-19 pandemic, following the 2020 survey. Furthermore, changes in health behaviors after the COVID-19 outbreak were analyzed by surveying changes in daily life (smoking, drinking, physical activity, skipping breakfast, and depressive mood).

This study used a question inventory consisting of three general characteristics items (educational stage, grade, and household economic status), two oral health behavior items (number of toothbrushing times per day, whether or not teeth were brushed after lunch), and two items on changes in everyday life due to COVID-19 (changes in drinking and smoking habits). Schools were divided into middle and high schools, and grades were divided into the first, second, and third grades of middle school and high school, respectively. Household economic status was divided into low-, medium-, and high-income groups. The number of toothbrushing times per day was categorized as ≤2 times, 3 times, ≥4 times, and the parameters related to whether students brushed their teeth after lunch at school in the past seven days were categorized as “no, I have not” and “yes, I have”, which is divided into “I always did”, “I mostly did”, and “I did sometimes”. Subjective health perception was divided into “healthy” and “unhealthy” with “very” and “somewhat” gradations for both. The factors for oral symptoms were “have received sealant treatment”, “have broken teeth”, “my teeth hurt when eating (not muscle pain)”, “have throbbing tooth pain”, and “have pain and bleeding in gums” with “yes” or “no” answers.

### 2.3. Data Analysis Methods

A complex sample design using stratification, clustering, and multistage sampling with weighting was employed to ensure a representative sample for the health behaviors survey. These weights were calculated by taking into account the sampling rates, response rates, and the demographic composition of the population. After multiplying the inverse of the sampling rate and the inverse of the response rate, the weights were adjusted to ensure that the sum of the weights for sex, school type (middle school, general high school, and specialized high school), and class matched the total number of middle and high school students nationwide as of April 2021. The steps involved in calculating these weights were as follows.

A weight was the product of inverse of sampling rate, inverse of response rate, and weight adjustment rate.

$$\mathrm{Weight}\;=\frac{1}{\mathrm{Sampling}\;\mathrm{rate}}\times \frac{1}{\mathrm{Response}\;\mathrm{rate}}\times \;\mathrm{Weight}\;\mathrm{adjustment}\;\mathrm{rate}$$

The sampling rate was calculated in reflection of the sampling process and was the product of the sample school extraction rate and sample class extraction rate.

The sampling rate was calculated as an inverse of sampling rate to adjust for sampling biases during population estimation.

$$\begin{array}{c} \frac{1}{\mathrm{Sampling}\;\mathrm{rate}}=\frac{1}{\mathrm{School}\;\mathrm{sampling}\;\mathrm{rate}}\times \frac{1}{\mathrm{Class}\;\mathrm{sampling}\;\mathrm{rate}}\\ =\frac{\mathrm{Number}\;\mathrm{of}\;\mathrm{schools}\;\mathrm{in}\;\mathrm{the}\;\mathrm{population}}{\mathrm{Number}\;\mathrm{of}\;\mathrm{sample}\;\mathrm{schools}}\times \;\mathrm{Number}\;\mathrm{of}\;\mathrm{classes}\;\mathrm{by}\;\mathrm{grade}\;\mathrm{in}\;\mathrm{the}\;\mathrm{sample}\;\mathrm{schools} \end{array}$$

For the response rate, the response rate by grade in the sample schools was employed, which was the percentage of subjects who participated in the survey from the total number of subjects by grade in the sample schools (number of students on the roll as of date of survey). The inverse of the response rate was calculated to ensure representation of the number of students who responded to the survey by grade.

$$\frac{1}{\mathrm{Response}\;\mathrm{rate}}=\frac{\mathrm{Number}\;\mathrm{of}\;\mathrm{subjects}\;\mathrm{by}\;\mathrm{grade}\;\mathrm{in}\;\mathrm{the}\;\mathrm{sample}\;\mathrm{schools}}{\mathrm{Number}\;\mathrm{of}\;\mathrm{respondents}\;\mathrm{by}\;\mathrm{grade}\;\mathrm{in}\;\mathrm{the}\;\mathrm{sample}\;\mathrm{schools}}$$

The weight adjustment rate was calculated such that the sum of the weights for sex, school type (middle school, general high school, and specialized high school), and class within the region matched the total number of middle and high school students nationwide as of April 2021.

$$\begin{array}{c} \mathrm{Weight}\;\mathrm{adjustment}\;\mathrm{rate}=\\ \frac{\mathrm{Number}\;\mathrm{of}\;\mathrm{students}\;\mathrm{by}\;\mathrm{sex},\;\mathrm{school}\;\mathrm{type},\;\mathrm{and}\;\mathrm{grade}\;\mathrm{in}\;\mathrm{the}\;\mathrm{region}\;\mathrm{of}\;\mathrm{the}\;\mathrm{population}}{\mathrm{Sum}\;\mathrm{of}\;\mathrm{weights}\;\mathrm{for}\;\mathrm{sex},\;\mathrm{school}\;\mathrm{type},\;\mathrm{and}\;\mathrm{grade}\;\mathrm{within}\;\mathrm{the}\;\mathrm{region}} \end{array}$$

Extreme weights were examined using the first quartile (Q1), third quartile (Q3), and the interquartile range (IQR = Q3 − Q1) of adjusted weights. Cases that deviated from the range (Q1 − 2 × IQR, Q3 + 2 × IQR) were defined as extreme weights. Weight trimming was performed, where extreme weights were replaced with either an upper bound (Q3 + 2 × IQR) or a lower bound (Q1 − 2 × IQR) weight value. Then, the weights were readjusted such that the sum of the weights matched the total number of middle and high school students nationwide as of April 2021.

According to the KDCPA guidelines for statistical analysis, the weighted values calculated by the KDCPA in 2021 were used to generate an analytical file. Frequency analysis was performed on participants’ general characteristics, oral symptoms, changes in drinking and smoking habits due to COVID-19, and subjective health perceptions due to COVID-19. Cross-tabulation analysis was performed to identify oral symptoms based on toothbrushing habits after lunch at school. Logistic regression analysis was performed to determine the relationship between oral symptoms and subjective health perceptions due to COVID-19. The IBM SPSS software (v. 22.0; IBM Corp., Armonk, NY, USA) was used to analyze the data, and statistical significance level was set at 0.05.

## 3. Results

### 3.1. General Characteristics and Oral Symptoms of Participants

General Characteristics and Oral Symptoms of Participants is shown in Table 1. Middle school students (13 ≤ age ≤ 15) accounted for 54.7% of participants, and high school students (16 ≤ age ≤ 18) accounted for 45.3%. Regarding economic status, nearly half were categorized as middle-income (49.4%) with only 11.3% in the low-income category. Regarding changes in everyday life due to COVID-19, the majority (83.6%) indicated “no change” in smoking habits (83.6%), and equally, a majority (81.1%) indicated “no change” in drinking habits. Regarding the number of toothbrushing times during the previous day, “3 times” (49.3%) and “4 times or more” (41.4%) accounted for most of the results. Regarding toothbrushing after lunch at school in the past seven days, the majority (59.1%) selected “no, I have not”.

Regarding oral symptoms in the past 12 months, 26.9% had undergone sealant treatment, 9.0% experienced broken teeth, 30.5% experienced tooth pain while eating, 20.9% experienced throbbing tooth pain, and 18.8% experienced pain and bleeding in the gums.

### 3.2. Subjective Health Perception Due to COVID-19

Subjective health perceptions due to COVID-19 among the participants are shown in Table 2. The results showed that 64.8% perceived they were “healthy”, 26.1% “moderate” in health, and 9.26% “unhealthy”.

### 3.3. Oral Symptoms Regarding Toothbrushing after Lunch at School

When students always brushed their teeth after lunch at school, the sealant treatment item showed a higher number of “no” (10.0%) responses compared to “yes” (4.1%; *p* < 0.001), and the broken teeth item also showed a higher number of “no” (13.0%) responses (“yes” = 1.2%; *p* < 0.001). Regarding tooth pain while eating, the number of participants who responded “no” (9.7%) was higher than those who responded “yes” (4.4%; *p* < 0.001), and regarding throbbing tooth pain, the number of “no” (10.9%) responses was higher compared to “yes” (3.2%; *p* < 0.001). A similar trend was observed for pain and bleeding in the gums (no [11.3%] vs. yes [2.8%]; *p* < 0.001). Refer to Table 3.

### 3.4. Relationship between Oral Symptoms and Subjective Health Perceptions Due to COVID-19

When students had no experience of sealant treatment due to COVID-19, responses indicating being “healthy” were higher (odds ratio [OR]: 1.06, 95% confidence interval [CI]: 0.99–1.13), and for the broken teeth item, “healthy” was also higher (OR: 1.30, 95% CI: 0.99–1.20); however, the difference was not significant.

Patients who reported no tooth pain while eating (OR: 0.65, 95% CI: 0.61–0.70), no throbbing tooth pain (OR: 0.64, 95% CI: 0.60–0.69), and no risk of pain and bleeding in the gums (OR: 0.63, 95% CI: 0.59–0.67) were perceived as healthy (*p* < 0.001). Refer to Table 4.

## 4. Discussion

This study was conducted on oral symptoms and subjective health perceptions in adolescents’ daily lives that have changed due to COVID-19.

Smoking refers to the act of aspirating nicotine through the mouth and nose, and it can cause various oral diseases such as discoloration of teeth and oral tissues, loss of taste, bad breath, hypersensitivity due to periodontal disease, delayed healing of wounds in the mouth, and oral cancer through direct contact with the mouth. Smokers have a higher number of bacteria in the mouth than non-smokers and an increased prevalence of periodontal destruction, so even if the same periodontal treatment is performed, the effectiveness of the treatment is much lower [10]. In particular, smoking and drinking alcohol in adolescence cause disorders in the liver, heart, and intestines, and affect the nervous system, even if the consumption is lower than that of adults, reducing judgment [11].

In a study of changes in college students’ health status due to the COVID-19 pandemic, drinking decreased in approximately 44% and increased in approximately 17% of respondents, whereas smoking both decreased and increased in approximately 17% of respondents compared to the pre-pandemic period [4]. Park and Lee [12] evaluated smoking and drinking in adolescents since COVID-19 and reported that it increased in approximately 1–3% of respondents, and decreased in approximately 13–15% of respondents. Similarly, we noted that drinking in adolescents increased in 2.8% of respondents and decreased in 15.5% since COVID-19, whereas smoking increased in 1.03% of respondents and decreased in 15.5%. This can be attributed to the shift to online classes due to COVID-19; adolescents stayed at home for longer and had less time to meet friends, leading to a decrease in drinking and smoking at higher rates than an increase in those habits. In cases where drinking and smoking decreased after COVID-19 rather than showing no change, the risk of tooth pain while eating and throbbing tooth pain was low, whereas in cases where drinking and smoking increased, the risk of pain and bleeding in the gums was high. Additionally, in cases where drinking increased, the risk of tooth pain while eating was also high [12]. Park and Kim [13] reported that adolescents who had a history of drinking and smoking had symptoms of oral disease at higher rates than those who had no such history. COVID-19 had a beneficial effect on lifestyle but possibly not on mental health.

Toothbrushing is the most basic and effective method for physically removing dental plaque to prevent the risk of dental caries and periodontal disease and maintain teeth and periodontal tissue health [14,15,16,17]. Treerutkuarkul and Gruber [18] recommend brushing teeth at least twice daily. Daily toothbrushing frequency is typically used as a variable representing individual oral health behaviors [19] and is an influential factor on dental caries and periodontal disease, and is closely related to health-risk behaviors [19,20]. In a study by Yeun et al. [21], the average number of toothbrushing times daily among participants was 2.42, which was categorized as twice or more per day, the minimum recommended level by the World Health Organization (WHO). The results indicate that most students in Callao to the north of Lima in Peru (87.9%) brushed their teeth twice or more per day; however, this value was lower than the average toothbrushing frequency of all adolescents in the Lima region in Peru [18,22]. The present study found that the number of times students brushed their teeth the previous day was “twice or less” for 9.3% of respondents, “3 times” for 49.3%, and “4 times or more” for 41.4%. When asked whether or not they brushed their teeth after lunch at school in the past seven days 59.1% responded “no, I have not”. Overall, the results indicate that students are following the rule of toothbrushing three times or more per day relatively well; however, the rate of toothbrushing after lunch was relatively low.

Regarding the relationship between oral symptoms and subjective health perceptions due to COVID-19, cases of no tooth pain while eating (OR: 0.65, 95% CI: 0.61–0.70), no throbbing tooth pain (OR: 0.64, 95% CI: 0.60–0.69), and no risk of pain and bleeding in the gums (OR: 0.63, 95% CI: 0.59–0.67) were perceived as healthy. Health-related knowledge, attitudes, and behaviors in adolescent students are affected by many risk factors inside and outside of school, and students’ health-risk behaviors lower their academic efficacy and have a negative impact on their school life, as well as threatening their physical health in adulthood [23,24]. Moreover, as adolescents’ oral health behaviors determine their future oral health, integrated approaches toward improving health risk and oral health behaviors among adolescent students are necessary [23,25].

Regarding behaviors such as smoking, drinking, engaging in physical activities, and dieting, developing a desirable lifestyle is crucial for preventing chronic diseases that are the leading causes of death in adulthood, such as cancer, heart disease, and cerebrovascular disease. Statistics showing the status and trends in health behaviors among adolescents in Korea generated from the KYRBS are utilized as important evidence for youth health policymaking, including the national health plan, as well as for international comparisons of youth health behaviors by international organizations such as the WHO and OECD.

The Ministry of Health and Welfare of the Republic of Korea states that enhancing toothbrushing education alone is not sufficient to induce positive behaviors in adolescents regarding toothbrushing. Instead, they emphasize the need for equipping schools—where adolescents spend most of their time—with effective toothbrushing facilities and equipment and enabling students to acquire self-practicable methods. This approach aims at ensuring that anyone in school can practice toothbrushing. However, in the current COVID-19 environment with the prevalence of online classes, students cannot brush their teeth at school, even when attending classes offline, and thus it is challenging to properly manage adolescents’ oral health. Now, the time has come to return to typical everyday life. Therefore, to practice and habituate the knowledge and behaviors of proper oral hygiene management through oral health education, it is important to reorganize the relevant facilities so that students can practice toothbrushing at school.

A limitation of this study is that the research tool consists of a self-filled questionnaire based on subjective experience of oral symptoms, so it is necessary to be cautious in using it as an objective indicator. Nonetheless, this study uses data that represent the association between oral symptoms and subjective health perception of adolescents that have changed due to COVID-19, and it is meaningful that it was studied in adolescents vulnerable to the effects of COVID-19.

## 5. Conclusions

This study used data representing Korean adolescents from the 17th KYRBS (2021) to investigate the relationship between oral symptoms and subjective health perceptions in adolescents due to COVID-19. It is significant that this study was conducted on adolescents vulnerable to the effects of COVID-19. One limitation is that the study item inventory consisted of self-administered survey questions on the subjective experience of oral symptoms; thus, it is necessary to be cautious when using them as objective indicators. Further studies are encouraged to improve the accuracy of the study tools by using objective clinical test results as indicators of oral symptoms.

The results of the study showed that students maintained an adequate level of oral hygiene but did not brush their teeth frequently after lunch. These results further revealed that COVID-19 may have influenced smoking and drinking behavior—which also affect oral health—in adolescents.

This study identified a relationship between oral symptoms and subjective health perceptions since the onset of COVID-19. Appropriate oral health management for adolescents is required due to the changes since COVID-19.

## Figures and Tables

**Table 1:** General characteristics of participants (*n* = 54,848).

Characteristics	Division	*n*	%
Educational stage	middle(13 ≤ age ≤ 15)	30,015	54.7
	high(16 ≤ age ≤ 18)	24,833	45.3
Grade	1st grade in middle school	10,016	18.3
	2nd grade in middle school	10,235	18.7
	3rd grade in middle school	9764	17.8
	1st grade in high school	8461	15.4
	2nd grade in high school	8647	15.8
	3rd grade in high school	7725	14.1
Economic status	high	21,568	39.3
	medium	27,077	49.4
	low	6023	11.3
Changes in everyday life due to COVID-19: smoking	increased	527	1.0
	no change	45,078	83.6
	decreased	8330	15.5
	total	53,935	100.0
Changes in everyday life due to COVID-19: drinking	increased	1518	2.8
	no change	44,659	81.8
	decreased	8425	15.5
	total	54,602	100.0
Number of toothbrushing times the previous day	twice or less	5079	9.3
	3 times	24,060	49.3
	4 times or more	25,709	41.4
Toothbrushing after lunch at school in the past 7 days	no, I have not	32,427	59.1
	yes, I have	22,421	40.9
Sealant treatment in the past 12 months	no	40,073	73.1
	yes	14,775	26.9
Broken teeth in the past 12 months	no	49,933	91.0
	yes	4915	9.0
Tooth pain while eating in the past 12 months	no	38,124	69.5
	yes	16,724	30.5
Throbbing tooth pain in the past 12 months	no	43,392	79.1
	yes	11,456	20.9
Pain and bleeding in gums in the past 12 months	no	44,526	81.2
	yes	10,322	18.8

**Table 2:** Subjective health perception since COVID-19 (*n* = 54,848).

Characteristics	Division	*n*	%
Subjective health perception	healthy	35,529	64.8
	moderate	14,298	26.1
	unhealthy	5021	9.2

**Table 3:** Oral symptoms based on toothbrushing after lunch at school (*n* = 54,848) chi-square test.

		Toothbrushing after Lunch at School				Total	*p*
		Always	Mostly	Sometimes	Never		
Sealant treatment	no	5511 (10.0)	3755 (6.8)	6879 (12.5)	23,928 (43.6)	40,073 (73.1)	<0.001
	yes	2248 (4.1)	1427 (2.6)	2601 (4.7)	8499 (15.5)	14,775 (26.9)	
Broken teeth	no	7126 (13.0)	4707 (8.6)	8512 (15.5)	29,588 (53.9)	49,933 (91.0)	<0.001
	yes	633 (1.2)	475 (0.9)	968 (1.8)	2839 (5.2)	4915 (9.0)	
Tooth pain while eating	no	5322 (9.7)	3627 (6.6)	6808 (12.4)	22,367 (40.8)	38,124 (69.5)	<0.001
	yes	2437 (4.4)	1555 (2.8)	2672 (4.9)	10,060 (18.3)	16,724 (30.5)	
Throbbing tooth pain	no	5978 (10.9)	4058 (7.4)	7654 (14.0)	25,702 (46.9)	43,392 (79.1)	<0.001
	yes	1781 (3.2)	1124 (2.0)	1826 (3.3)	6725 (12.3)	11,456 (20.9)	
Pain and bleeding in gums	no	6219 (11.3)	4176 (7.6)	7753 (14.1)	26,378 (48.1)	44,526 (81.2)	0.024
	yes	1540 (2.8)	1006 (1.8)	1727 (3.1)	6049 (11.0)	10,322 (18.8)	

**Table 4:** Relationship between oral symptoms and subjective health perception due to COVID-19 (*n* = 40,550) logistic regression.

	Subjective Health Perceptions (Unhealthy = 0, Healthy = 1)		
	OR	95% CI	*p*
Sealant treatment (no = 0/yes = 1)	1.06	0.99–1.13	0.103
Broken teeth (no = 0/yes = 1)	1.09	0.99–1.20	0.098
Tooth pain while eating (no = 0/yes = 1)	0.65	0.61–0.70	<0.001
Throbbing tooth pain (no = 0/yes = 1)	0.64	0.60–0.69	<0.001
Pain and bleeding in gums (no = 0/yes = 1)	0.63	0.59–0.67	<0.001

R² = 0.016, adjusted R² = 0.034, F = 5.00, *p* < 0.001.

## Data Availability

The datasets used and/or analyzed during the current study are available from the corresponding author on reasonable request.
